# Teaming up to traverse loneliness: a co-creative journey toward a home care work model for supporting social participation among older adults

**DOI:** 10.1186/s12913-022-08524-y

**Published:** 2022-09-14

**Authors:** Therese Nordin, Anna-Britt Coe, Ingeborg Nilsson

**Affiliations:** 1 Department of Community Medicine and Rehabilitation Units: Occupational Therapy, Umea University, Umeå, Sweden; 2 Department of Sociology, Umea University, Umeå, Sweden

**Keywords:** Home care services, Participatory action research, Older adults, Social participation, Togetherness, Occupational therapy, Elderly care

## Abstract

**Background:**

Participatory research is particularly suitable in adressing know-do gaps in health systems. There is a disparity between what is known about the benefits of social participation and home care’s responsibility to provide conditions amenable to older adults’ social participation, and what is accomplished in home care practice. Home care workers are a large, low-power group, whose competences should be better harnessed. We carried out a participatory action research (PAR) project with the goal of generating an improved structure for identifying and alleviating loneliness. This article aims to explore the co-creative process of designing a work model that guides home care workers in supporting social participation among older care recipients.

**Methods:**

Multimodal data from 16 PAR workshops with 14 home care workers were described and explored through the ‘recursive PAR process’ and the ‘framework for occupational enablement for change in community practice”.

**Results:**

The PAR process is outlined through the objectives, activities, and work model, as well as enablement strategies employed throughout the PAR process; as are its opportunities, challenges and implications. The work model describes how care workers can act as discoverers of care recipients’ unmet social needs, employ intentional communication, and link to relevant professions or community services to alleviate loneliness among older home care recipients.

**Conclusions:**

This research process included opportunities of collaborating with enthusiastic and competent home care workers, but also challenges of moving between theory and practice and maintaining active participation between workshops. The resulting work model is in step with the requirements of elderly care, is unique in its field and could comprise a first step toward a more systematic approach of assessing and addressing loneliness. The vivid delineation of the PAR process provided in this paper can aid other researchers in navigating participatory research in home care contexts.

**Supplementary Information:**

The online version contains supplementary material available at 10.1186/s12913-022-08524-y.

## Background

Participatory styles of research has been suggested to be particularly suitable in adressing know-do gaps in health systems [1], and including issues relevant to women [2]. Participatory action research (PAR) acts within a specific context and focuses on social action and change [3]. Scientists and stakeholders work together to examine problems and generate context-specific solutions, even when stakeholders lack experience in research or organizatitional development [4]. This collaborative generation of knowledge is often called ‘co-creation’ [5]. Central to co-creative and participatory processes are fostering mutual respect, capacity-building and empowerment [4], and uncovering tacit knowledge and competencies. Various creative methods such as role playing, storyboarding, and futures workshops are often utilized [3, 6].

To address a know-do gap in a complex context with workers with vast practical knowledge but low formal education, we carried out a PAR process striving to improve structures for identifying and alleviating loneliness. This article aims to explore the co-creative process of designing a work model that guides home care workers in supporting social participation among older care recipients.

Loneliness and social isolation are increasingly acknowledged for causing ill health [7, 8, 9, 10, 11]. Older adults are more likely to face reduced leisure activities [12] and social networks, and, consequently, to experience loneliness [13]. The opportunity to participate in society and to maintain individually-relevant relationships; i.e. ‘social participation’, is a key component in better health and wellbeing [14, 15, 16]. However, loneliness and social participation are complex experiences and situated in day-to-day life, and with assistance in daily activities the situation becomes even more multifaceted.

In Sweden today, like in many other countries, home care is the most common form of elderly care [17]. More than half of Swedish home care recipients report feeling lonely ‘sometimes’ or ‘often', [18] which has remained fairly constant over recent years. Research has shown that older home care recipients perceives agency in managing interests and relationships as important for satisfactory social participation [19], and yet other research has indicated that home care workers can both facilitate and hinder care recipients’ own decicions [20]. The Social Services Act [21] regulates home care, and although it stipulates that older adults have a right to assistance in engaging in a meaningful life with others, systematic approaches for assessing and adressing social needs are lacking [22]. Evidently, there seems to be a gap between what is known about the benefits of social participation and home care’s responsibility to provide conditions amenable for recipients to engage in a meaningful life with others, and what is accomplished in practice: a so-called “know-do gap” [1].

Swedish home care services encompass at-home support for community dwelling persons, regarding for example household tasks, personal care, medical care and emotional/social support [23], by assistant nurses. All Swedish home care services are financed with public funding, and provided by either municipal or private organizations [23]. The applicant’s care needs are assessed and potentially granted by municipal home care assessors, and if the municipality also has private options available, the care recipient may choose their provider. This system aims to increase older adults opportunities to excert choice and control, but research have shown it in some cases can be counter productive and increase dependency and experiences of lack of control in daily living [24]. While the time slot and formal content is strictly delineated in service grants, the individual care worker is rather alone in deciding *how* to carry out the service. Delivering home care support is complex [25], marked by restraints on time, working alone, and balancing conflicting values [26, 27]. This demanding work situation has been described as one of low control, affecting care workers’ health, quality of work life, and their output quality of care [28]. But while stress of conscience and exhaustion are common, home care workers often describe their jobs as meaningful and morally fulfilling [29]. And while the profession is known for its complexity, home care workers have low levels of formal education; usually assistant nurse training (training at a high school level), but a lack of formal care training is also common. This contributes to the profession’s low status, low salary levels and low power. Employment in the elderly care sector (home care, care homes and home health care) comprises the largest employment sector in Sweden, where 90% of care workers are women [30] and 25% of employees in the care and service sector were born in another country.

Research examining Swedish home care workers’ perspectives on supporting social needs is sparse. A discourse analysis showed that home care workers value social support for care recipients and that their obligations and opportunities could involve both strengthening their current procedures or developing structures to better fit the social needs of older home care recipients [31]. Research from other countries shows that care workers can have a positive attitude towards supporting meaningful and social activities [32], but that physical care is often seen as home care’s main concern and the lack of time, knowledge and awareness of such issues remain barriers [33]. Therefore, it has been argued that addressing organizational factors might be crucial in shaping conditions to enhance a socially-oriented and person-centered approach to elderly care [26].

## Methods

### Study design

This project had a PAR approach [3], focusing on co-creating knowledge with home care organizations in an attempt to improve support for social participation among home care recipients. PAR [3] was chosen for its recursive orientation towards action and change in practice. Furthermore, the study was inspired by ‘participatory design’ [6] and ‘futures methodology’ [34, 35] for their respective focuses on designing prototypes and providing a concrete structure for people without design experience to examine problems and constructing a model for change.

### Roles in the collaborative PAR-process

Concurrent with a participatory ontology [36], participants were viewed as situational experts on home care and on work model content. The researchers’ role included creating space, breaking down the end goal into manageable steps, providing evidence-based knowledge on loneliness and social participation, and supporting operationalization of home care workers’ competencies.

## Reflexivity and researcher-as-instrument

The collaborative relationships in PAR requires an active and reflexive researcher; the researcher-as-instrument [37]. We attempted to consciously use ourselves [38] to balance power and facilitate a collaborative space; for example through purposive adaptability in verbal, emotive, written, spatial and material communication. This requires awareness of, for example, our backgrounds and preconceptions and transparency in the report [37]. The first and last authors, who led the workshops, have a background in occupational therapy, and the second author, who functioned as PAR methodology expert, is a sociologist. Whereas second and third authors are experienced researchers with PhD degrees, first author was a doctoral student. Occupational therapy includes philosophical assumptions [39], for example, viewing people as autonomous and with the potential for participation and as the driving force of their own change, which guided our facilitation of the PAR process. Additionally, all authors has previous work experience in elderly care and rehabilitation.

### Context

This project, “Stay In Touch”, is part of a multi-disciplinary research program, “Future Care” [40], where three universities, in multiple projects, collaborate with healthcare to increase social participation among elder care recipients. The Stay In Touch project is generating knowledge about loneliness among older adults in a home care context in several studies [19, 31].

Umea University is a comprehensive university in northern Sweden, located in proximity to the participating home care organizations. The municipalities were relatively small and semi-rural, and did not have private home care options.

### Recruitment and participants

As a PAR process requires a prolonged committment, we aimed to recruit organisations and participants with a strong interest in the challenge of loneliness. After a public presentation by IN in a local newspaper, two home care managers made contact and volunteered to participate. In turn, IN and TN gave another presentation at each of the care organizations’ regular staff meetings where all employees are required to attend, informing care workers of the project and inviting them to participate. We emphasized seeking all ages, genders, and levels of experience, thereby striving for varied groups. All who volunteered were included in the study and provided informed consent.

**Fig. 1:**
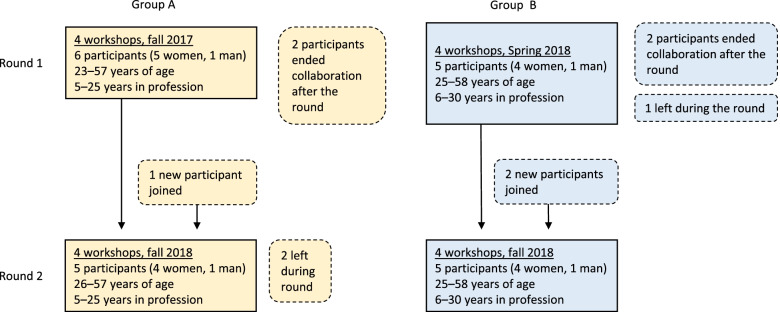
Flow chart of recruitment and participants

We first recruited participants for four workshops in each organization, and 11 home care workers chose to participate. Those participants were later invited to a second round of four workshops, in which seven participants decided to extend their participation, and three new participants joined the project. In total, 14 home care workers (equally distributed from care organizations A and B) participated in the study (Fig. 1). The two groups covered a wide range of ages (23–58) and years of experience in home care (5–30). Most participants were women, and most had a high school education, with additional courses at high school or college level. One participant was racialized. Three participants decided to adjourn their participation before the end of a round, and expressed a heavy workload, not interested in the development process, or gave no explanation as to their withdrawal.

### Data generation and analysis

After establishing collaboration with the care managers, IN and TN accompanied a care worker in their daily work for 2 days each, to gain an understanding of their work situation.

The co-creation process consisted of a total of 16 workshops: three sub-cycles which together amounted to one over-arching PAR cycle (Fig. 3). Each workshop took place about once per month, for 2.5 hours, at the home care organizations’ respective office buildings. Initially, eight workshops were planned (first round), and the latter eight workshops (second round) were added upon need. The PAR process generated a vast amount of data, including summaries, field notes, mind maps, textual and graphical drafts, audio recordings (~ 12 hours) and videos, and a list of data, workshop topics, action-oriented research questions is provided in Additional file 1: Appendix 1. The final Stay In Touch model included a five-step figure and chart (Fig. 3). After the workshops ended, researchers prepared material for pilot testing (a website, mobile application and introductory material) which was reviewed and verified by two volunteering participants.

The data analysis proceeded in two phases. The first phase was a hands-on process that proceeded continuously with data generation, through reflecting upon data to direct the next action. In practice, researchers discussed and summarized data, which the participants then departed from when developing the work model further. This process generated questions; for example “what can social activities encompass?”, which in turn raised new questions, as they were examined through action-oriented modalities. In the beginning, these questions were primarily introduced by researchers, and as the process matured, they became more participant-driven. The analytical process also encompassed monitoring and facilitating a fruitful group climate.

The second phase of analysis consisted of scrutinizing data to explore the co-creative process. First, all data (for example workshop plans, audio files, sketches, field notes) were reviewed and sorted in chronological order. Through iterative examination of the data (Additional file 1: Appendix 1), objectives and activities of each workshop were extracted and descriptive text for each sub-cycle was formulated. These descriptions were then related to McIntyres “recursive process of PAR” [3] and Rensburgs “framework for occupational enablement” [41], for the purpose of highlighting and interpreting the group process, researchers’ enabling strategies, and obstacles and opportunities. This analysis was mainly performed by TN, and intersperesed with extensive reflection among all authors throughout the analysis process.

## Results

**Fig. 2:**
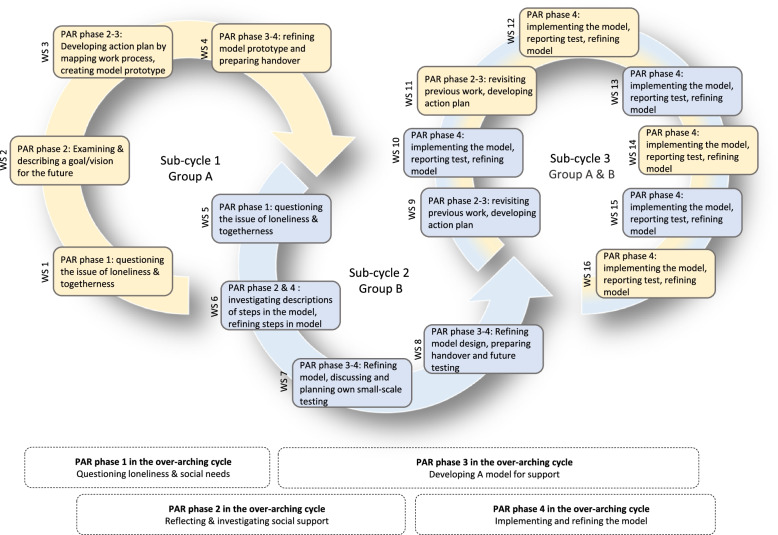
The process of PAR workshops. Each circle represents a sub-cycle, and all together they make up the over-arching PAR cycle. Yellow represents group A and blue represents group B. Each box represents a workshop (WS)

In line with PAR, the process is a part of the result and thus described in this section. The three PAR sub-cycles that together made up one over-arching PAR cycle (see Fig. 2) is addressed in chronological order, and lastly the created work model is presented.

All of the *enablement foundations* and *facilitators of enablement* described by Rensburg [41] were utilized in the overarching PAR cycle, but they varied in pertinence over the course of the PAR process. At the beginning of each sub-cycle, all participants were provided a folder with information and writing material, to use as they liked. All workshops were structured with a warm-up phase, a working phase, and an ending phase. Ongoing engagement was also expected between workshops, such as reflecting on a specific part of the model in their daily work.

### Questioning the present and envisioning an alternative future: first round of workshops

This round consisted of two sequential sub-cycles of workshops with similar layouts. Both sub-cycles included reflecting upon loneliness among care recipients, envisioning a “social future” for older care recipients and then modeling a concrete plan for change (structure inspired by Futures workshops [34]). The creative assignments used to explore topics and questions were inspired by creative group work and process mapping [42], to stimulate reflection and operationalization.

Since the project and relationships was in its initial stages, two of the “enablement foundations” [41] were particularly relevant: creating a ‘shared vision of possibilities’ and exploring how ‘change, transformation, liberation, and actualization’ could come about. In addition, two “facilitators of enablement” [41] were often utilized in the first round: using intentional ‘communication’ and ‘fostering relationships’. In the interdependent relationship between researchers and participants, researchers took on a relatively active role in introducing objectives and activities.

### Generating an initial model of change with group A (sub-cycle 1)

This sub-cycle corresponded to the first phase of the overarching PAR cycle: reflecting upon loneliness among care recipients and investigating how social participation could be supported within the existing organization of home care services. The sub-cycle also included the initial steps of development of an action plan and the “implementation” of this plan into a first model draft.

#### Objectives and activities

The objective of group A’s four workshops was to generate an initial work model. Firstly, the participants reflected on their own social lives through mind-mapping. After a short lecture about research on older adult loneliness, participants explored their perceptions of older care recipients’ loneliness and social needs (problem formulation). Family, friends, home care workers and people of the same age were described as important for providing support to older care recipients to do social activities. Hinders included lack of strength, feeling nervous or being uninformed about local events. They summarized their discussion as follows:

“*Maybe it isn’t so important what you do, but that you get to go outside your home, see something else, meet people. They are missing someone to encourage them and come along, maybe the first time. Tell them about upcoming activities for older people*”

The group then created a fictive care recipient for whom they envisioned a rich social life through drawing, discussing and writing. The participants mapped current work procedures and examined how those could be enhanced to identify, address, and evaluate social needs. They sketched a five-phase pie-chart structure to depict their enhanced work process. They also wrote a short description and fictive case example for each phase. Finally, the participants made a video presenting their draft and formulated suggestions for further development for the next group.

#### Facilitators of enablement and group climate

The assignments were intentionally concrete to make the process manageable, and encouraging communication were carefully exercised to foster positive relationships. The participants seemed enthusiastic toward the matters at hand, and worked relatively independently and goal-oriented during the work-phases. The suggested questions and assignments seemed to fit the participants’ competencies and successfully contribute to the positive and creative climate. Reflecting on own social preferences produced subsequent discussions on care recipients’ variations in social needs. Creating a fictive care recipient concretized the visionary discussion, which has been previously described as well [43]. Participants showed confidence and competence in discussing the concrete details of supporting social participation, whereas mapping and abstracting these competences proved more challenging, which was reflected when discussions became tangential and their progress slowed down.

### Further developing the initial model of change with group B (sub-cycle 2)

This round corresponded with the middle phases of the overarching PAR cycle: continuing with developing the plan and initializing implementation and refinement.

#### Objectives and activities

The objective of group B’s four workshops was to refine and informally test group A’s initial model. This group began with examining their perceptions of older care recipients’ social situation and envisioning a positive future through brainstorming and collaging. They reviewed group A’s problem and vision formulations, model draft and video, and discussed how well this fit their own perceptions of the problem and visions. They then reflected on facilitators and hinders of each phase, the model’s overall feasibility. For example, a challenge that was discussed was care recipients’ lack of knowledge about their rights regarding services that home can provide, and they emphasized the value of information. One participant explained an example of lack of information like this:

“*they don’t know that they can request … what’s it called … well a staff member comes along to the city for a day, to go shopping with them. Many don’t know that*”

The group also developed changes and additions. The model’s descriptive text was elaborated, a symbolic color scheme and a symbol for person-centeredness was added, and pie-chart design was changed into a circle of action points with an additional inner circle depicting a smaller process. The participants also attempted to plan informal testing between the third and fourth workshop. Last, the group identified conditions important for further testing (Table 1), and prepared a video presenting their refined model.

**Table 1:** Important conditions when testing the model in a larger scale, as identified by participants

Important for future testing	
➢Managers’ involvement in the decision to implement is crucial	
➢Some extra time is needed in the beginning, to develop the frame of mind [få in tänket]	
➢Try to integrate the work with existing structures and tools, such as recurrent quality of care-meetings	
➢Potentially using the work phone to increase the model’s accessibility in daily work	
➢Collegial discussions in small groups, about how to do it in practice, and preferably using case examples that sparks imagination and comprehension	
➢Documentation of actions done in the Stay In Touch process are crucial	
➢Language matters; wordings in the model, in home care assessor grants [biståndsbeslut] and in direct communication with care recipients. Loneliness can be a sensitive issue that requires a delicate approach, and the standardised wording of case manager grants can be difficult to understand for care recipients.	

Conditions identified by the participants as important to consider when testing the model in a larger scale. Hyphens [x] represents original wording in Swedish. The translation to English was done for this article with the support of a professional language editor

#### Facilitators of enablement and group climate

It quickly became clear that the sequential setup of this round (i.e. taking over another group’s work) gave group B a more challenging start. Also, two out of the five participants were not able to attend the first workshop, which prolonged the group’s formation process. Participants showed engagement and competence, but independent work proved more challenging in this sub-cycle, especially regarding abstraction of their practice-based knowledge. Therefore, the researchers changed approach and participated more actively: interdependence and collaborative planning and doing became the prominent strategies. A successful approach became the preparation of visual concretizations; i.e. making several design examples that captured the participants’ previous discussions. This strategy supported critical reflection and sparked creative thinking.

Initially, sub-cycle 2 was intended to encompass informal testing of the model, but the participants needed all four workshops to reach a model they were comfortable with. The suggestion to prepare small scale testing between third and fourth workshop was received with caution, and ultimately, little testing was carried out. Researchers concluded that testing would require additional preparation, and therefore, both groups were invited to another round of workshops.

### Testing and refining the model: second round of workshops

This round consisted of two parallel sub-cycles of workshops. In the over-arching PAR cycle, this round corresponded with the last phase: implementing and refining the plan. Although ‘communication’ and ‘fostering relationships’ remained important, the relationships between researchers and participants felt relatively established, and ‘collaborative planning’ and ‘monitoring the process’ became more pertinent facilitators of enablement. The researchers increased encouragement for participants to take on a more active role in the interdependent relationship. Towards the end of the round we also developed ‘strategies for sustainability and handover’.

### Testing and refining the model with groups A and B simultaneously (sub-cycle 3)

In this cycle, the two groups worked simultaneously and transferred suggestions, questions, and changes through the researchers. This aimed to align the groups’ preconditions and enable a sense of community. The participants tested the work model in everyday home care work between workshops, and a typical workshop began with a participant-moderated reporting session (introduced to increase feelings of ownership), followed by refining details in the work model according to needs discovered during testing.

#### Objectives and activities

The objective of this round was to test and refine the model and produce case examples. Both groups’ initial tasks encompassed making a plan for the workshops and for testing the model in practice. Group A decided to focus on the small-scale process (‘here and now’), due to their restrained work situation, and group B decided to strive for three full-scale examples and one small-scale example. Both groups wished to receive reminding weekly text-messages. Transfers between groups concerned problematic areas and/or suggestions for changes to text and graphics. They discussed, for example, how to enable planned activities in practice, through verbal and non-verbal communication:

- Group A: “*you have to show engagement in the activity, that’s what’s needed*”
- Group B: “*you need to act inviting in some way*”

Communication with other professionals, such as home care assessors was also identified as a potential threshold, and was thus emphasized more strongly in the model. One participant described how power differences can affect care recipients’ expression of needs to different professions:

“*it can become another type of conversation between the care assessor and the care recipient. [---] often, I talk a little differently to the care recipient, we get sort of a closer relationship, but when the care assessor comes, it’s almost like with the nurse: ‘no no everything is just fine’*”

The testing process resulted in 24 case examples of varied completeness, and the work model was elaborated with more explicit delineations of person-centeredness and care recipient agency, and was more strongly tied to existing structures like care meetings and the contact care worker’s responsibilities. They also formulated questions for identifying loneliness and wishes for specific support, need for documentation, and discussed and determined a Swedish name for the work model: “Håll kontakten”.

#### Facilitators of enablement and group climate

In this round, one of the groups had a new manager, which seemed to alter the groups’ mood: participants appeared down-hearted, but were nonetheless engaged in the process. In both groups, testing still seemed somewhat difficult to grasp, why researchers prepared note-taking booklets, containing spaces for noting phases of concern, description of actions, and experienced challenges and opportunities. The researchers’ attempts to encourage participant leadership sometimes generated insecurities rather than empowerment. The participant moderation of test reports worked unevenly: while some adopted the task with confidence, others seemed insecure. To meet these insecurities, researchers strived to convey availability and support without taking over. The preparation of concrete summaries and design alternatives continued to be successful strategies for enabling creative and critical thinking. Weekly text messages were described as helpful for remembering and prioritizing testing among their regular work tasks.

### The stay in touch work model

The over-arching PAR cycle, consisting of three sub-cycles, resulted in a work model called ‘Stay In Touch’ [‘Håll kontakten’]. It describes how home care workers can, within the boundaries of their role, act as discoverers of unmet social needs, employ intentional communication, and link to other professions in order to facilitate more person-centered support for social participation among older care recipients. The model can, in a way, be seen as a frame of mind, which illuminates loneliness and social support within in the regular organization of home care and provides guidance in day-to-day contact with care recipients.

**Fig. 3:**
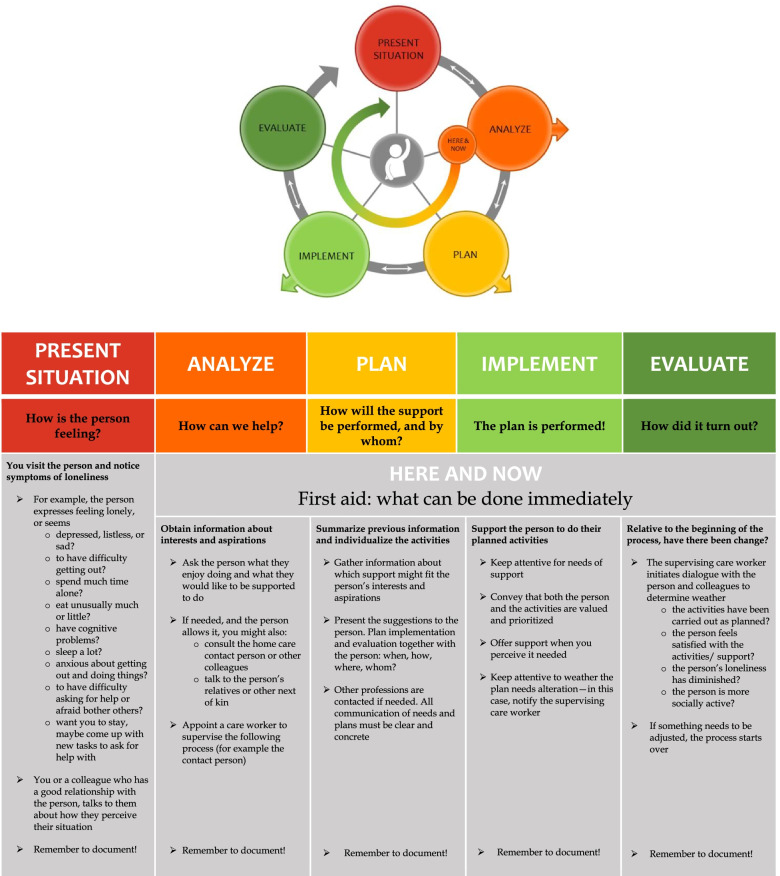
Stay In Touch Circle and Chart. This chart is a detailed description of the content of each phase in the Stay In Touch Process, formulated by the participants. The original chart was done in Swedish and translation to English was done for this article with the support of a professional language editor

The Stay In Touch model consists of a process of five phases, depicted as a large circle with an additional inner circle and a chart describing each phase (Fig. 3). Symbols were carefully chosen by participants to convey, for example, iteration, early withdrawal and person-centeredness, and the traffic-light color scheme symbolizes the process moving from a bad to a good situation. Being attentive, responsive, encouraging, adaptive, and exercising professional judgement are strategies emphasized in the participants’ description of how to employ the Stay In Touch process. The model describes actions from the care worker’s position, but participants were adamant about the care recipient’s agency, which is mirrored in their formulations in the chart.

The model begins with phase *Present situation,* and conveys importance of being attentive to signs of loneliness and using ordinary small talk to learn if the person experiences problems with loneliness (or referring this task to another care worker). If the person confirms loneliness, phase *Analyze* follows. The inquiry continues by asking what the care recipient enjoys doing, which relationships they value, and if they think home care could provide support. The care worker can also, with the care recipient’s approval, discuss potential support with colleagues or the person’s next of kin. One care worker will be assigned to monitor the process, preferably the ‘contact care worker’. Phase *Plan* includes examining potential support to suggest to the care recipient. In this phase, colleagues or other relevant professions can be involved with the care recipient’s approval. The phase might require application for additional service grants, in which case, a home care assessor will perform planning. However, it is also possible that the care recipient’s aspirations fit within existing grants (such as social stimulation, meal company, or walks), and planning can be done informally or via structures for individual care planning. Clear communication between different professions is emphasized. In phase *Implement*, the care worker’s role depends largely on the result of previous phase, but emphasizes using judgement and showing that the person’s chosen activities are valued and prioritized. The last phase, *Evaluate,* encompasses dialogue with the care recipient and home care colleagues, and distinguishes four aspects of evaluation. Depending on the outcome, the process can be closed or start over at a suitable phase.

The model also contains an inner circle, *Here & Now*, which represents a shorter series of (informal) actions that reflects a small-scale Stay In Touch process that can be done immediately. For example: a person expresses feeling lonely (present situation) and longing for a relative (analyze), the home care worker asks/suggests a telephone call (plan) and help finding and dialing the number (implement) and the person seems satisfied for the moment (evaluate).

## Discussion

The activities in this process led to achieving it’s goal of generating an improved structure for identifying and alleviating loneliness among home care recipients, and this paper explores this co-creative process. However, the journey was not without its challenges. PAR is well known for being an unpredictable and time-consuming research style [3], and reaching a tested and refined final version that participants were satisfied with, required an additional round of eight workshops. Participants struggled with both abstracting their knowledge, and putting their abstraction back into practice. The researchers’ strategies to meet these struggles and facilitate an affirmative and progressive group climate were illuminated through the ‘enablement foundations’ and ‘facilitators of enablement [41]; in the secondary data analysis of this article. This framework has, to our knowledge, never been used to guide or analyze a PAR process before, and proved useful in supporting understanding of our enabling process.

The testing phase required extensive reminding and encouraging participants to keep the project in mind between workshops and to prioritize their planned testing, and the researchers had initially aspired for a more exhausted testing. Similar experiences have been described in other PAR processes [3]. A possible explanation for this engagement-drop might be the well-known precursors in home care contexts: stress [26, 27] and low focus on social issues [22, 33]. Such preconditions will likely hamper engagement in adding social tasks (even when innately valued) when they are competing with more strongly incited tasks and values. Therefore, creating preconditions where care recipients’ social well-being is formally acknowledged as a home care concern, is likely an important aspect of successful implementation of the Stay In Touch model.

Power dynamics within the project could also have affected the participants’ engagement. It is well known that PAR processes are susceptible to power imbalances [3], and that this can decrease feelings of meaningfulness and active participation [41]. ‘Power sharing’ is one of the central foundations in the enablement model [41], and throughout the process, we strived to flatten power and empower participants to feel ownership. This was, however, rather challenging: when the researchers attempted to move to the background, participants often expressed unease. Similar dilemmas have been previously described [3, 44], along with the need for a fluid shift between participants’ and researchers’ ‘expert’ perspectives. McIntyre [3] describes how expectance to reflect and take responsibility often generates anxiety, and that joint responsibility can require extended support and time. Rensburg [41] delineates importance of participants’ opportunities to define objectives, plan activities and evaluate their engagement. We strived to utilize these values within the inevitable project boundaries and our suggested activities and objectives aimed to crystallize the participants’ views *within* this frame. This orchestration contributed to the model progress, but might also have hampered feelings of ownership. It is possible that even more time together could have enabled naturally occurring doldrums to proceed and eventually resolve into consensus and empowerment. It was, however, not possible to extend the time frame beyond what we already had done, but the importance of a large and flexible time frame should be considered for future research.

Manager involvement is another aspect that might have impacted participant engagement. One of the groups had different managers in first and second round, which gave us an opportunity to meet the same participants under two leaderships. The former manager had initiated participation in the project, whereas the latter expressed lower priority of the same. In this shift, the group’s atmosphere changed visibly from strongly enthusiastic to more muted engagement, and two participants in this group also decided to end their participation before the end of the round. Leadership involvement and support have been identified as crucial for engagement and change when care workers participate in research, both by current participants (Table 1) and in other research [45]. The possibility of increasing manager involvement was repeatedly discussed among researchers during the co-creation process, and we held verification meetings with the managers before and after each round. But in retrospect, it might have been beneficial if managers had also been involved during workshops in some way, in order to support empowerment within the power dynamics of the organization. Rensburg [41] describes the importance of involving all relevant stakeholders, which became clear in the current project, but it is also evident that effectively applying this in practice is a challenge. One way to support identification of stakeholders, power structures, and change-relevant positioning, might be to perform a power analysis of the organization [46, 47] prior to the project or in collaboration with participants.

Despite struggles, participants identified a way to support social participation through existing structures of home care. Their produced model corresponds well with Swedish social services’ foundational values for elderly care; for example, emphasizing care recipients’ rights to a meaningful existence with others, respect for care recipient’s autonomy, and capitalizing on the care recipient’s own social resources [21]. It also fits well with the required competencies for staff in elderly care [48], which emphasizes supporting social participation, adaptive communication with care recipients, their relatives, and relevant professions, and documentation in the patient journal. Interestingly, participants identified communication with other professions as a potential barrier, particularly that a difference between the lay-language used when talking about a care recipient’s needs and the formal wording of written home care grants sometimes caused care recipients to decline granted support. Participants also identified care recipients’ insecurities as possible hindrances during the implementation phase, and they accentuated the importance of care workers conveying that they value and prioritize the care recipients’ chosen activities. Another interesting trait of the participants’ model is their explication of tacit knowledge in general and the small-scale process *Here and Now* which positions the smaller, “extra” tasks, done while doing other tasks, as an important part of meeting social needs.

To our knowledge, this is the first co-created work model for supporting social participation in a home care context. It must, of course, be tested and validated on a larger scale, but it offers a first step toward increasing systematic approaches in assessing loneliness and addressing social needs within the vast and complex context of home care. Likely, such testing would identify opportunities for further refinement, which is an expected and positive continuation of participatory action research.

### Methodological discussion

This paper attempted to provide vivid descriptions of the PAR process, our facilitative approaches, and the opportunities and challenges met during the journey. Home care workers match the type of vulnerable and low-power populations that PAR was developed to reach, but paradoxically, low power also brings challenges in the research process and might limit the potential for change [3]. Throughout the collaboration, the researchers strived to learn about the context, through for example auscultations and engaging in casual conversation during snack breaks. The researchers’ summaries and examples nourished critical reflection among participants and sparked both rejection and elaboration of elements in the work model, as did the testing in practice. Reflexivity between the researchers was cultivated through de-briefing sessions where facilitating approaches were scrutinized (for example about how to arrange the room, carry themselves, and stepping back or stepping forward). These discussions were immensely valuable, and in hindsight, they could have been audio-recorded to provide further insights for secondary analysis.

There were relatively few drop outs, but three participants left the collaboration before the end of a round, which could be considered a limitation. The researchers’ impression was, however, that their adjournment did not cause (or was caused by) friction in the groups. The slight change in the groups’ constellations between first and second rounds could be viewed both as a limitation and a strength. The majority remained, which allowed a continuation in group development, while the few newcomers provided appreciated perspectives. All participants and home care managers who initiated collaboration expressed interest in improving social support for care recipients, which is crucial for successful PAR research and was a strength in the PAR process.

## Conclusions

This paper describes and analyzes a participatory action research process, where home care workers and researchers collaboratively created a work model that aims to guide home care workers in supporting social participation among older care recipients. This paper explores the research process’s intrinsic objectives, activities, and facilitators of enablement, and discusses opportunities and challenges. We met challenges including maintaining active participation between workshops and participants struggling with moving between theory and practice, and empowerment where participants felt ownership of the process and the model seemed unfulfilled. Nonetheless, the project also contained opportunities for engaging home care workers who demonstrated competence in their field and provided opportunities to highlight their tacit knowledge, describe ways to traverse care recipients’ loneliness and support social participation within the boundaries of the home care worker’s roles. The participants identified potential barriers for example regarding communication between professions and addressed it in their work model. The produced work model resonates well with the foundational values and skills required in elderly care, and voices that tacit knowledge of experienced home care workers. The model is unique in its kind, and could comprise a first step toward a more systematic approach to assessing and addressing loneliness in the home care context.

The vivid delineation of the PAR process as well as its challenges and opportunities that is provided in this paper, can aid other researchers in navigating participatory research in home care contexts.

## Supplementary Information

**Additional file 1: Appendix 1.**

## Data Availability

The datasets generated and/or analyzed during the current study are not publicly available, because the data includes information that could compromise research participants’ privacy and consent but are available from the corresponding author on reasonable request.

## Abbreviation

PAR: Participatory Action Research
