# Supplementary material for: Teaming up to traverse loneliness: a co-creative journey toward a home care work model for supporting social participation among older adults
Source: BMC Health Serv Res. 2022 Sep 14;22:1159. doi: 10.1186/s12913-022-08524-y (PMC9476274; doi:10.1186/s12913-022-08524-y)
Supplement: Supplementary file 1 — Additional file 1: Appendix 1. [file 12913_2022_8524_MOESM1_ESM.docx]

|  | | | Topics and ACTION ORIENTED research questions (RQ) | Researcher-produced data | Participant-produced data |
| --- | --- | --- | --- | --- | --- |
| First round: sequential questioning, reflecting and developing a model design | Sub-Cycle 1, Fall 2017 | WS 1  GRP A | **PAR phase 1: questioning the issue of loneliness & togetherness**  *RQ: What can loneliness and social activities encompass?* | - WS-plan - Instructions for assignments - ML booklet: research on loneliness - Field notes | - Mind map: own valued relations & activities - Mind map: care recipients’ loneliness situation - Text: problem formulation - Audio: 85 min |
|  |  | WS 2  GRP A | **PAR phase 2: Examining & describing a goal/vision for the future** *RQ: How should/could social participation be for older care recipients?* | - WS-plan - Instructions for assignments - ML booklet: research on social participation | - Visual: Avatar symbolizing older care recipients - Mind map: social vision for care recipients - Text: goal formulation/vision - Audio: 105 min |
|  |  | WS 3  GRP A | **PAR phase 3: Developing action plan by mapping/sequencing work process and creating a model prototype**  *RQ: How can we work in practice, to move towards the goal?* | - WS-plan - Instructions for assignments - ML booklet about process models | - Mind maps: identifying, addressing, evaluating loneliness in HC - Sequential figure of above activities - Text: Practical example of each step - Audio: 55 min |
|  |  | WS 4  GRP A | **PAR phase 4: refining prototype model prototype and preparing handover**  *RQ: Do our draft cover our thoughts so far, and what does next group need for continuing to build the model?* | - WS-plan - Instructions for assignments - Clean copy of model draft - Diploma | - Text: description of content in each step - Visual: Final model prototype - Video: presenting the model - Questions/instructions for next group |
|  |  |  | **Meeting: Verification with manager A**  *RQ: Does the model prototype align with home care services’ boundaries and preconditions?* | - Summarizing PP |  |
|  | Sub-Cycle 2, Spring 2018 | WS 5  GRP B | **PAR phase 1: questioning the issue of loneliness & togetherness**  *RQ: what can loneliness and social activities encompass*  *RQ: In which ways do we concur or disagree with the former group’s prototype?* | - WS-plan - Instructions for assignments - ML booklet: loneliness, social participation and previous group’s problem & goal | - Mind map: problem and goal exploration |
|  |  | WS 6  GRP B | **PAR phase 2 & 4 investigating descriptions of steps in the model, refining steps in model**  *RQ: How can we develop the process description to match what we think is important when supporting social participation?* | - WS-plan - Instructions for assignments - ML booklet: process modelling - Examples of various model types | - Text: refined content of steps in model - Visual: refined graphics in model - Audio: 105 min |
|  |  | WS 7  GRP B | **PAR phase 3-4: Refining model design, developing implementation plan by discussing and planning own small scale testing**  *RQ: What needs to be done to create preconditions for testing?*  *RQ: How do I perform my testing?* | - WS-plan - Instructions for assignments - Clean copies of model variations | - Text: Bullet points important for testing - Text: Individual plans for informal testing - Visual: Refined model - Audio: 77 min |
|  |  | WS 8  GRP B | **PAR phase 3-4: Refining model design, discussions on testing, preparing handover**  *RQ: What did we learn from small scale individual testing?*  *RQ: What is important for making pilot-testing work locally?*  *RQ: What do we want to transfer to the next group?* | - WS-plan - Instructions for assignments - ML: information about next step in project - Visual: diploma - Field notes | - Mind map of test report - Video: presenting the model - Audio: 46 min |
|  |  |  | **Meeting: Verification with manager A**  *RQ: Does the model prototype align with home care services’ boundaries and preconditions?* | - Summarizing PP |  |
| Second round: parallel testing and refining the model | Sub-cycle 3, Fall 2018 | WS 9  GRP B | **PAR phase 2-3: revisiting previous work, developing action plan**  *RQ: How do we want to go about testing the model in daily work?*  *RQ: How do we want to structure the four workshops?*  *RQ: How do we create case examples?* | - WS-plan - ML: recap of project & previous work - Matrix for test plan - Field notes | - Mind map: how to proceed with testing - Test plan - Audio: 22 min |
|  |  | WS 10,  GRP B | **PAR phase 4: implementing the model, reporting test, refining model**  *RQ: How do we make the text more linguistically consequent and person-centered?*  *RQ: How do we know when to initiate the SIT-process? RQ: How do we depict early withdrawal?* | - WS-plan - Customized booklet for documenting testing - Instruction for participant moderator - Reminding text-message - Field notes | - Visual/test: changes in step description in model - Audio: 48 min |
|  |  | WS 11 GRP A | **PAR phase 2-3: revisiting previous work, developing action plan**  *RQ: How do we want to go about testing the model in daily work?*  *RQ: How do we want to structure the four workshops?*  *RQ: How do we create case examples?* | - WS-plan - ML: recap of project & previous work | - Audio: 49 min |
|  |  | WS 12 GRP A | **PAR phase 4: implementing the model, reporting test, refining model**  *RQ: How can we create a “toolbox” for social stimulation?*  *RQ: How do we fortify the “contact staff member” in the SIT-process?* | - WS-plan - Instruction for participant moderator - Reminding text-message - Field notes | - Notes for group B - Audio: 77 min |
|  |  | WS 13  GRP B | **PAR phase 4: implementing the model, reporting test, refining model**  *RQ: How can we create a “toolbox” for social stimulation?*  *RQ: How do we fortify the “contact staff member” in the SIT process?*  *RQ: How do we gather information and offer support?* | - WS-plan - Instruction for participant moderator - Printed models of comparing versions - Reminding text-message - Field notes | - Text/visual: printed model with notes in pencil - Audio: 77 min |
|  |  | WS 14  GRP A | **PAR phase 4: implementing the model, reporting test, refining model**  *RQ: How do we introduce a person responsible for monitoring the process?*  *RQ: How do we highlight staff engagement throughout the SIT process?*  *RQ: Step 3 plan could become a threshold; how do we prevent that?*  *RQ: What can evaluation mean within the boundaries of the home care worker’s role?* | - WS-plan - Instruction for participant moderator - Comparison of A’s and B’s versions - Reminding text-message - Field notes | - Text/visual: printed model/steps with notes in pencil - Audio 94: min |
|  |  | WS 15 GRP B | **PAR phase 4: implementing the model, reporting test, refining model**  *RQ: What do we want to call the model?*  *RQ: How can we create recorded case examples?*  *RQ: Which final adjustments do we want to do?* | - WS-plan - Instruction for participant moderator - Reminding text-message - Field notes | - Booklets with documented tests - Text/visual: printed model with notes in pencil - Audio: 46 min |
|  |  | WS 16  GRP A | **PAR phase 4: implementing the model, reporting test, refining model**  *RQ: What do we want to call the model?*  *RQ: How can we create recorded case examples?*  *RQ: Which final adjustments do we want to make?* | - WS-plan - Instruction for participant moderator - Visual: Summary of all changes made - Reminding text-message | - Booklets with documented tests - Text/visual: printed model with notes in pencil - Audio: 95 min |
|  |  |  | **Meeting: Verification with manager B**  *RQ: Does the model align with home care services’ boundaries & preconditions?*  *RQ: If and how the care organization wants to continue with pilot testing?* | - Summarizing PP |  |
|  |  |  | **Meeting: Verification with manager A**  *RQ: Does the model align with home care services’ boundaries and preconditions?*  *RQ: if and how the care organization wants to continue with pilot testing?* | - Summarizing PP |  |
| Additional collaboration | | Grp A&B | Case descriptions of testing, 4 participants |  | - Audio: 7 recordings á 2-8 min, total: 37 min |
|  |  | GRP B | Verifying additional material, 2 participants | - Implementation plan, documentation matrix, mobile phone application and web page |  |
